# Leaf Physiological and Proteomic Analysis to Elucidate Silicon Induced Adaptive Response under Salt Stress in *Rosa hybrida* ‘Rock Fire’

**DOI:** 10.3390/ijms18081768

**Published:** 2017-08-14

**Authors:** Prabhakaran Soundararajan, Abinaya Manivannan, Chung Ho Ko, Sowbiya Muneer, Byoung Ryong Jeong

**Affiliations:** 1 Institute of Agriculture and Life Science, Gyeongsang National University, Jinju 660-701, Korea; prabhakaran.s.bioinfo@gmail.com (P.S.); abinayamanivannan@gmail.com (A.M.); 2 Division of Applied Life Science (BK21 Plus), Graduate School, Gyeongsang National University, Jinju 660-701, Korea; tune0820@gmail.com (C.H.K.); sobiyakhan126@gmail.com (S.M.); 3 Institute of Life Science, Gyeongsang National University, Jinju 660-701, Korea

**Keywords:** hydroponics, rose, resistance, salinity, silicate

## Abstract

Beneficial effects of silicon (Si) on growth and development have been witnessed in several plants. Nevertheless, studies on roses are merely reported. Therefore, the present investigation was carried out to illustrate the impact of Si on photosynthesis, antioxidant defense and leaf proteome of rose under salinity stress. In vitro-grown, acclimatized *Rosa hybrida* ‘Rock Fire’ were hydroponically treated with four treatments, such as control, Si (1.8 mM), NaCl (50 mM), and Si+NaCl. After 15 days, the consequences of salinity stress and the response of Si addition were analyzed. Scorching of leaf edges and stomatal damages occurred due to salt stress was ameliorated under Si supplementation. Similarly, reduction of gas exchange, photosynthetic pigments, higher lipid peroxidation rate, and accumulation of reactive oxygen species under salinity stress were mitigated in Si treatment. Lesser oxidative stress observed was correlated with the enhanced activity and expression of antioxidant enzymes, such as superoxide dismutase, catalase, and ascorbate peroxidase in Si+NaCl treatment. Importantly, sodium transportation was synergistically restricted with the stimulated counter-uptake of potassium in Si+NaCl treatment. Furthermore, two-dimensional electrophoresis (2-DE) and matrix-assisted laser desorption/ionization time-of-flight mass spectrometry (MALDI-TOF MS) results showed that out of 40 identified proteins, on comparison with control 34 proteins were down-accumulated and six proteins were up-accumulated due to salinity stress. Meanwhile, addition of Si with NaCl treatment enhanced the abundance of 30 proteins and downregulated five proteins. Differentially-expressed proteins were functionally classified into six groups, such as photosynthesis (22%), carbohydrate/energy metabolism (20%), transcription/translation (20%), stress/redox homeostasis (12%), ion binding (13%), and ubiquitination (8%). Hence, the findings reported in this work could facilitate a deeper understanding on potential mechanism(s) adapted by rose due to the exogenous Si supplementation during the salinity stress.

## 1. Introduction

Salinity is one of the major abiotic stresses that limits the growth and development of plants [1]. In plants under salt stress, uptake of water and ion, photosynthesis, respiration, and other metabolic processes are deeply affected. A deficit in water uptake followed by ionic imbalance causes oxidative, ionic, and osmotic stress [2,3]. Impairment in the photosynthetic process leads to the higher lipid peroxidation and excessive accumulation of reactive oxygen species (ROS), such as superoxide anion (O_2_^−^) and hydrogen peroxide (H_2_O_2_) [4]. Though ROS are the by-products of vital metabolisms the built-in antioxidant system maintains the ROS under the controlled level. Temporal- and spatial-localization of ROS is vital for the regulation of signaling mechanisms [5]. Highly-accumulated ROS, due to decreased gas exchange processes and impairment in protective mechanism(s) could damage the cellular components, such as lipids, proteins, and nucleic acids [6]. Initially, superoxide dismutase (SOD) is involved in the dismutation of O_2_^−^ into H_2_O_2_. Further reduction of H_2_O_2_ into H_2_O and O_2_ are mediated by the catalase (CAT), peroxidases (POD), and other enzymes [4,5,6]. Plants possessing efficient antioxidant systems retain more tolerance against oxidative stress [7].

Though roses are one of the top ranking floricultural crops in ornamental plant trade, most of the rose varieties are sensitive to salinity. Therefore, the cultivation of roses is highly hindered by salt stress [8]. A previous report suggested that leaf injury occurred in *R. chinensis* ‘Major’ and *R. rubiginosa* even at 20 or 30 mM NaCl treatment [9]. Likewise, high concentrations of salt for short-term application resulted in defoliation and/or plant death [10,11]. Growth of rose rootstocks, such as *R. hybrida* L. ‘Dr. Huey’, *R.* x *fortuniana* Lindl., *R. multiflora* Thunb., and *R. odorata* (Andr.) ‘Sweet’ were deeply affected under saline conditions [12]. Other subspecies/varieties, such as *Rosa* X spp. L ‘Bridal White’ and ‘Red France’ grafted on *R. hybrida* ‘Manetti’, *R. odorata* (Andr.) ‘Natal Briar’, and ‘Dr. Huey’ showed decreased biomass, cut flower production, and foliage quality during salinity stress condition [13]. Meanwhile, silicate supplementation increased the cut flower quality of miniature rose ‘Pinocchio’ [14]. Moreover Si addition enhanced resistance against black spot disease [15] and powdery mildew [16] in ‘Meipelta’.

Silicon (Si) is the second most abundant element in the Earth’s crust [17]. The International Plant Nutrition Institute recently included Si as a ”quasi-essential” element. Application of Si increased tolerance against various abiotic [18,19,20] and biotic [21,22] stresses in most plants. Physiological improvements due to Si are associated with an enhanced leaf area, improved light interception, and elevated net photosynthetic assimilation [23]. On one hand, amorphous Si deposited in the leaf reduces the transpiration rate [24]. On the other hand, the accumulation of Si in the root promotes hydraulic movement and facilitates selective ion uptake [25]. Furthermore, previous studies reported that, during oxidative stress, Si assisted in the maintenance of redox homeostasis [7]. Detoxification of ROS in the Si treatment was associated with the modulation of the activities of antioxidant enzymes [26,27,28]. Higher accumulation of Na^+^ and Cl^−^ ions during saline conditions hinders the availability of essential elements in the plants [2,3,4]. While the amelioration effects of Si under salt stress have been intensively studied in many plants [18,19,20,25,26,27,28], only few reports are available in roses [29,30].

Particularly, no study has described the leaf proteomic changes in rose under any certain circumstances. Proteomic approaches offers a platform to unveil the pathways-shift associated with the physiological responses [31,32]. Transcriptomics or proteomics analysis allows unearthing the dynamic range of detailed mechanism(s) in plant system. Information on the tolerance developed against the stress obtained from the proteomic study ultimately supports the crop improvement strategies. In order to gain deeper insight into Si-induced salt tolerance, two-dimensional electrophoresis (2DE) and mass spectrometry (MS) have been employed.

Therefore, the current study was carried out to determine the role of Si on the leaf physiology, antioxidant metabolism, and proteome under salinity stress in the hydroponically-grown *R. hybrida* ‘Rock Fire’. To our knowledge, this is the first study describing the leaf proteomic changes in rose.

## 2. Results

### 2.1. Physiology and Photosynthesis

**Figure 1:**
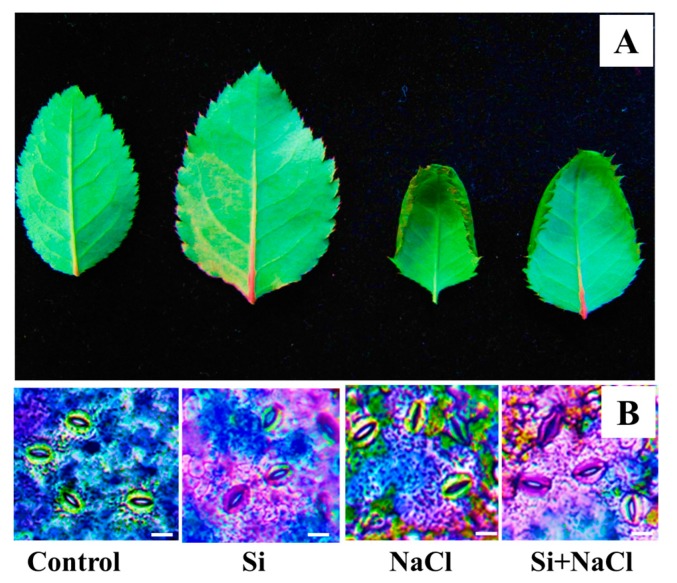
Leaf morphology (**A**) and stomatal structure (**B**) of *Rosa hybrida* ’Rock’ Fire in response to NaCl stress with and without silicon (Si) supplementation after 15 days. Bars: 100 µm.

**Figure 2:**
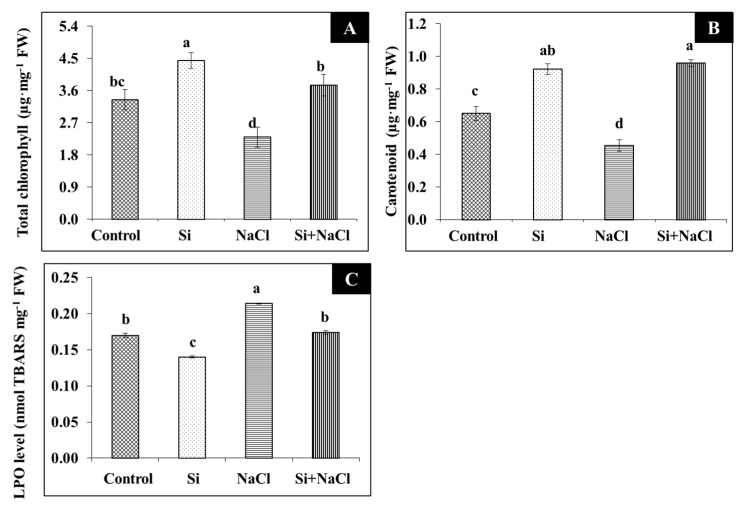
Photosynthetic pigments (total chlorophyll (**A**), carotenoid (**B**)) and lipid peroxidation (LPO) (**C**) of *Rosa hybrida* ‘Rock Fire’ in response to NaCl and Si supplementation. Data are the mean ± standard deviation (SD) from three replicates. Different letters indicate that treatments are significantly different at *p* ≤ 0.05.

**Figure 3:**
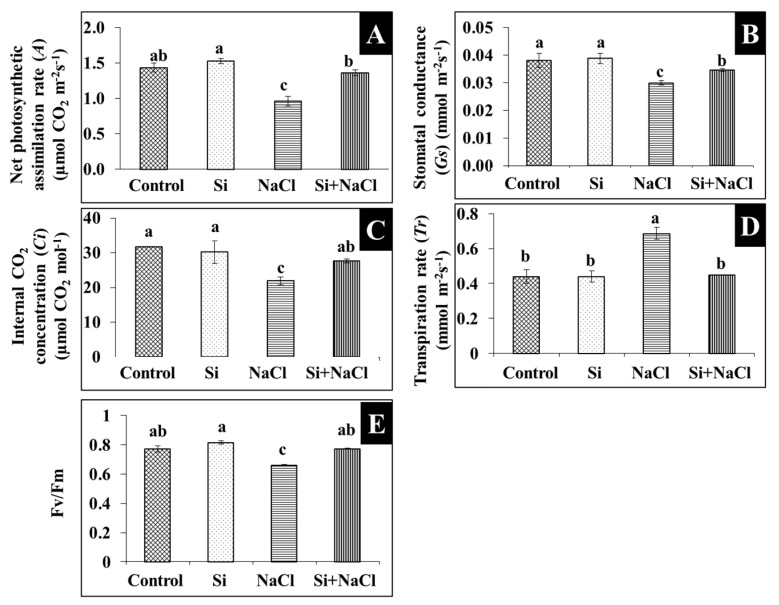
Photosynthetic gas exchange parameter analysis. Net photosynthetic assimilation (*A*) (**A**), stomatal conductance (*Gs*) (**B**), intercellular CO_2_ concentration (*Ci*) (**C**), transpiration rate (*Tr*) (**D**), and maximum quantum efficiency of PSII (*F*v/*F*m) (**E**) in fully-expanded penultimate leaves of *Rosa hybrida* ‘Rock Fire’ in response to NaCl stress with and without Si supplementation after 15 days. Data are the mean ± SD from three replicates.

Scorching and shriveling of leaves were observed in *R. hybrida* ‘Rock Fire’ under salinity stress was at least partially prevented by the Si treatment (Figure 1A). Similarly, stomatal development affected due to salt accumulation was mitigated under Si supplementation (Figure 1B). Impairment on the gas exchange related-parameters such as net photosynthetic assimilation rate (*A*), stomatal conductance (*Gs*), internal CO_2_ concentration (*Ci*), transpiration rate (*Tr*), and quantum efficiency (F*v*/F*m*) are directly correlated with the decrease in the content of photosynthetic pigments, such as chlorophyll (Figure 2A) and carotenoid (Figure 2B) during salt stress. Meanwhile, improvements in the synthesis of photosynthetic pigments (Figure 2A,B) are associated with enhanced gas exchange in Si+NaCl treatment (Figure 3).

### 2.2. Oxidative Stress and Analysis of Antioxidant Enzymes

**Figure 4:**
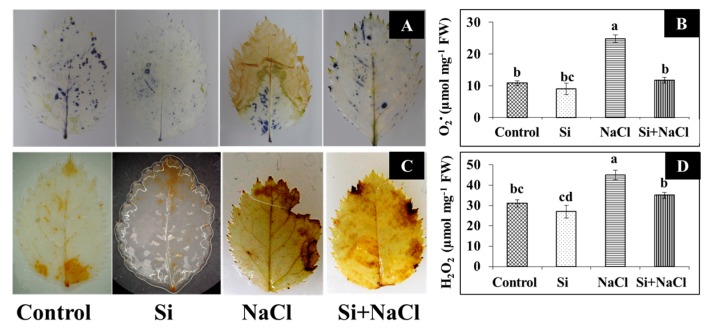
Histochemical localization (**A**,**C**) and content (**B**,**D**) of O_2_^−^ and H_2_O_2_ in the leaves of *Rosa hybrida* ‘Rock Fire’, respectively, during salt stress and Si treatments. Blue spots indicate the O_2_^−^ localized (**A**) and brown spots indicate the H_2_O_2_ (**C**) in the leaves. Data are the mean ± SD from three replicates. Different letters indicate that treatments are significantly different at *p* ≤ 0.05.

**Figure 5:**
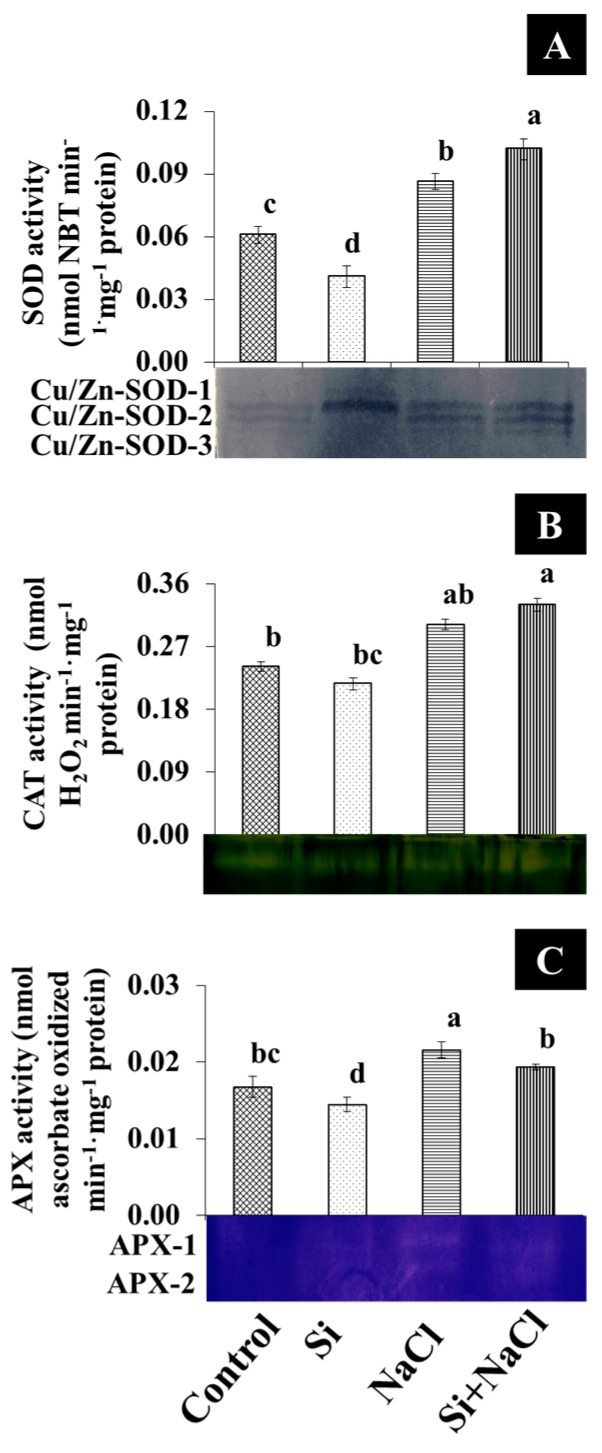
Activity and isozymes pattern of antioxidant enzymes such as superoxide dismutase (**A**), catalase (**B**), and ascorbate peroxidise (**C**) under salt stress with or without Si supplementation. Data are the mean ± SD from three replicates. Different letters indicate that treatments are significantly different at *p* ≤ 0.05.

**Figure 6:**
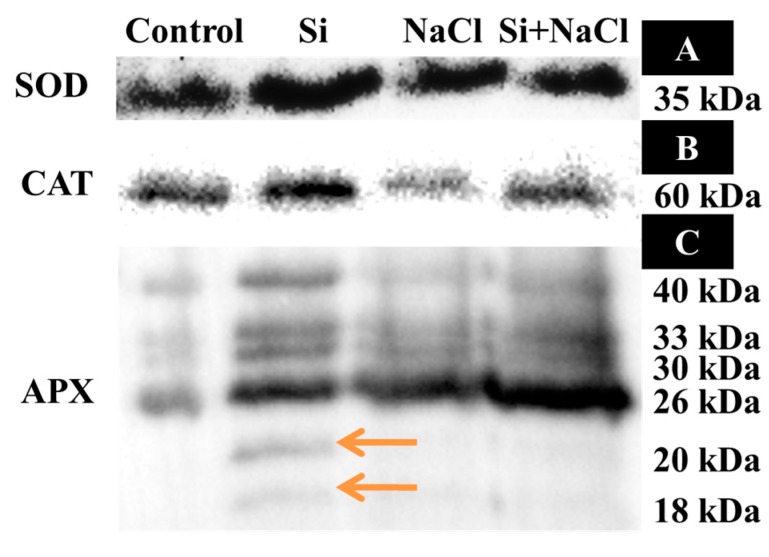
Immunoblotting analysis of superoxide dismutase (SOD) (**A**), catalase (CAT) (**B**), and ascorbate peroxidase (APX) (**C**). Differentially-expressed isomers of APX are denoted with orange arrows.

Although a higher lipid peroxidation (LPO) rate was observed in NaCl treatment, the addition of Si along with NaCl efficiently decreased the amount of malondialdehyde (MDA) (Figure 2C). Consequently, excessive accumulation of ROS, such as O_2_^−^ (Figure 4A,B) and H_2_O_2_ (Figure 4C,D), were observed upon salinity stress. However, augmentation of Si alleviated the disproportionate generation and detoxification of ROS. Among the antioxidant enzymes, SOD activity was higher in the Si+NaCl treatment (Figure 5A). However, the immunoblot results showed no significant difference between NaCl and Si+NaCl treatments (Figure 6A). Stimulated activity of CAT by Si under NaCl stress (Figure 5B) correlated with the expression of CAT isomers (Figure 6B). On the other hand, activity of ascorbate peroxidase (APX) increased as the NaCl treatment was decreased upon the presence of Si (Figure 5C). Supplementation of Si under normal conditions induced the differential expression of APX isomers (Figure 6C).

### 2.3. Uptake of Silicon, Sodium, and Potassium

**Figure 7:**
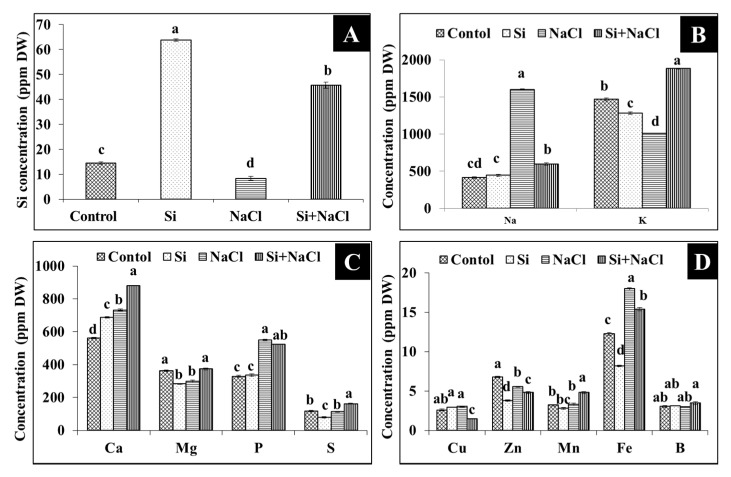
Content of silicon (**A**), sodium and potassium (**B**), and other macro- (**C**) and micro- (**D**) nutrients in response to salt stress with or without Si supplementation. Data are the mean ± SD from three replicates. Different letters indicate that the treatments are significantly different at *p* ≤ 0.05.

Accumulation of Si in the leaves of *R. hybrida* ‘Rock Fire’ was 63.84 µg·g^−1^ dry weight (Figure 7A), whereas the content of Si was decreased to 45.66 µg·g^−1^ dry weight in the Si+NaCl treatment. Most importantly, Na content in the Si+NaCl treatment was reduced 62.75% than in the NaCl treatment. Consequently counter-uptake of K against Na was observed in the Si-supplemented NaCl treatment (Figure 7B). Likewise, Si and/or NaCl influenced the other macro-(P, Ca, Mg, and S) (Figure 7C) and micro-(Zn, Fe, Mn, B, and Cu) (Figure 7D) element levels in the plant.

### 2.4. Dynamic Changes in the Expression of Proteins

**Figure 8:**
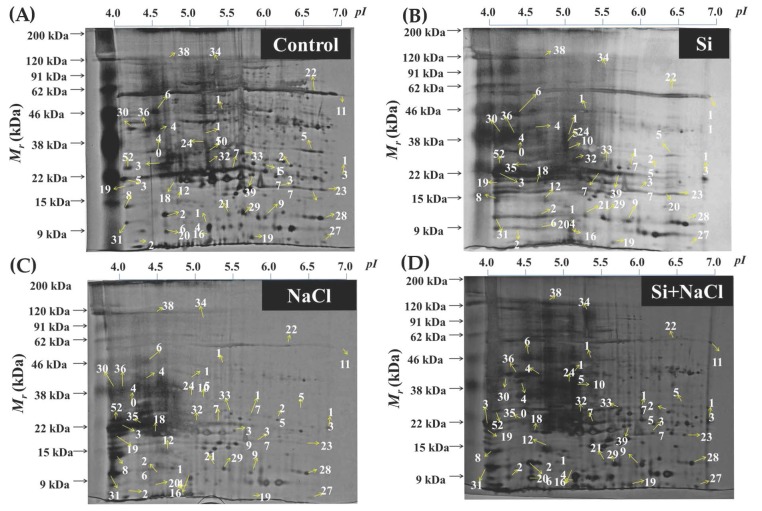
Representative images of two-dimensional gel electrophoresis (2-DE) gel of proteins extracted from the leaves *Rosa hybrida* ‘Rock Fire’ of control (**A**), Si (**B**), NaCl (**C**) and Si+NaCl (**D**) treatments, respectively. Differentially-expressed spots excised for the identification of the proteins by matrix-assisted laser desorption/ionization time-of-flight mass spectrometry (MALDI-TOF MS) are marked by the arrows.

**Figure 9:**
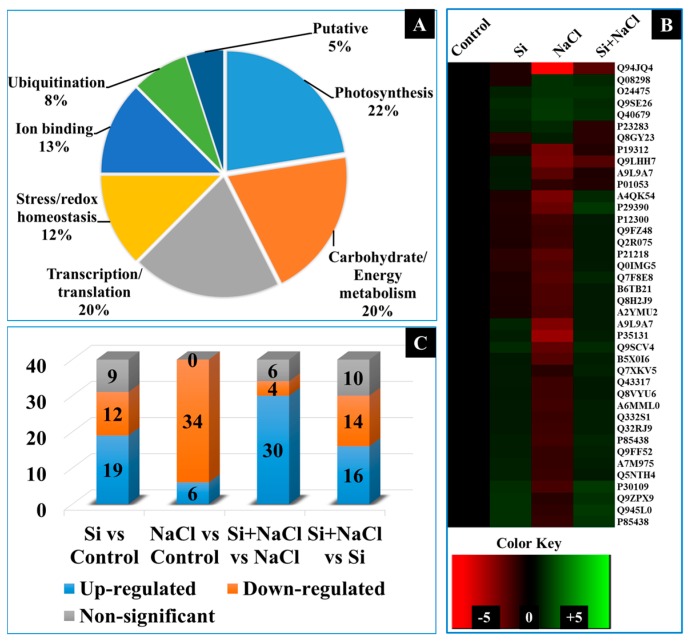
Comparative analysis of proteomic profiles between the treatments. Functional classification of identified proteins by Gene Ontology analysis (**A**), Heat map representation of differentially-expressed proteins (**B**), and the Venn diagram illustration of up-, down-, or non-significantly regulated proteins (**C**).

A total of 120 protein spots displayed more than 2.5-fold difference in abundance between the treatments. Among them 40 protein spots were identified using matrix-assisted laser desorption/ionization time-of-flight mass spectrometry (MALDI-TOF MS) analyses (Figure 8). The spot abundance and functional categorization of the identified protein spots were listed in Table 1. The identified proteins were functionally classified into six groups, such as photosynthesis (22%), energy metabolism (20%), transcription/translation (20%), stress/redox homeostasis (12%), ion binding (13%), and ubiquitination (8%)-related proteins (Figure 9A). A heat map illustration represented in Figure 9B displays the differential expressions of identified proteins in Si, NaCl, and Si+NaCl treatments with respect to the control (Figure 9B). In detail, in comparison with the control, Si alone treatment increased the levels of 19 proteins spots; meanwhile, the abundance of 12 proteins were decreased, respectively. On the other hand, 34 spots were downregulated and six spots were upregulated under salinity stress. Simultaneously, the addition of Si to NaCl treatment enhanced the abundance of 30 proteins and the expression of six protein spots were retarded in comparison with the NaCl treatment (Figure 9C).

#### 2.4.1. Photosynthesis-Related Proteins

The salinity stress decreased the expression of vital proteins involved in photosynthesis, such as NAD(P)H-quinone oxidoreductase subunit H (spot 7) and J (spot 24), ferredoxin-NADP reductase (spot 14), cytochrome c oxidase subunit 6b-2 (spot 12), photosystem I assembly protein Ycf4 (spot 26, 33, and 35), ribulose bisphosphate carboxylase SSU5B (RuBisCO) small chain (spot 28), and protochlorophyllide reductase B (spot 31). However, the supplementation of Si positively influenced the abundance of the abovementioned proteins and, thus, restored the photosynthesis process in the salt-stressed plants.

#### 2.4.2. Proteins Related to Energy Metabolism

Enzymes involved in the hydrolysis of carbohydrates, such as β-glucosidases (spot 11), β-galactosidases (spot 18), and glucose-1-phosphate adenylyltransferase large subunits (spot 30), were down-accumulated in the NaCl treatment. However, the amendment of Si increased the levels of carbon metabolism associated proteins (spot 11, 18, and 30). Similarly, acetyl-CoA carboxylase (spot 13 and 21), a precursor enzyme for fatty acid synthesis and glycerol-3-phosphate dehydrogenase (GPDH) (NAD^+^) (spot 32), a major linking enzymes between the carbohydrate metabolism and lipid biosynthesis were decreased by the NaCl treatment was improved upon the Si supplementation. On the contrary, isocitrate lyase (spot 3) and bifunctional protein FolD 2 (spot 27) protein spots increased in the salt stressed plants were decreased upon the Si addition.

#### 2.4.3. Proteins Related to Transcription and Translation

Proteins associated with translation and transcription such as 50S ribosomal protein L14 (spot 20), 60S ribosomal protein (spot 32), and CTR9 homolog (spot 40) were highly affected by the NaCl treatment. Moreover, decrease in the amino acid biosynthesis-related proteins, such as reactive intermediate deaminase A (spot 5), cysteine synthase (spot 39), and shikimate kinase 1 (spot 36), were observed under NaCl treatment. Similarly, proteins involved in the final process of amino acid biosynthesis, such as ribosomes recycling factor (spot 14) and tRNA(Ile)-lysidine synthase (spot 17), were also downregulated under the NaCl treatment. Proteins related to transcription/translation were induced upon the Si supplementation in *R. hybrida* ‘Rock Fire’.

#### 2.4.4. Proteins Involved in Redox Homeostasis

The abundance of glutathione s-transferases (GSTs) (spot 15) protein suppressed in the NaCl alone treatment was enhanced by the inclusion of Si. Consequently, the peroxygenase (spot 24) enzyme induced in NaCl treatment was decreased in the Si+NaCl treatment. Meanwhile, the levels of proteins responsible for the external stress stimuli (desiccation-related protein PCC3-06 (spot 10) and (dehydration-responsive protein RD22 (spot 23)) induced by the salt stress were reduced by the supplementation of Si.

#### 2.4.5. Proteins Involved in Ion Binding

Upregulation of proteins related to ion binding, such as Kinesin-like calmodulin binding protein (KCBP)-interacting Ca^2+^-binding protein (KIC) (spot 2), anamorsin homolog (spot 7), Ferritin-2 (spot 40), and metallothionein-like protein 4A (spot 39), were observed in both Si treatments under normal and stressed condition except the pinene synthase (spot 36).

#### 2.4.6. Proteins Involved in Ubiquitination

Though, E3 ubiquitin-protein ligase (spot 41) was downregulated by the Si treatment, other key proteins for ubiquitination, such as ubiquitin-conjugating enzyme E2 8 (spot 4), and E2 36 (spot 44), were upregulated in the Si+NaCl treatment.

## 3. Discussion

From this study, it is evident that the addition of Si improved the tolerance against salinity stress in *R. hybrida* ‘Rock Fire’. Increased leaf area (Figure 1) enhances the light interception [23]. It helps to maintain an upright growth to improve the CO_2_ assimilation and photosynthetic rate. Progressive development in the stomata (Figure 1B) could have assisted in the proper gas exchange process (Figure 2). In agreement with the present results, augmentation of Si on stomatal growth and gas exchange-related parameters against NaCl stress were reported in *Capsicum annum* [19]. Likewise, hardness provided by Si attributed for the protection of photosynthetic pigment and to maintain an appropriate water status [24].

A higher amount of MDA produced in the NaCl treatment (Figure 3C) indicated elevated LPO and released a very high amount of free radicals due to cell membrane damage (Figure 4). Generally, in abiotic stress conditions, the production of ROS are much higher than its detoxification [4]. Several reports suggested that supplementation of Si enhanced the activities of antioxidant enzymes to scavenge the excessively-produced ROS under stress conditions [18,19,26,27,28]. Constitutive involvement of Si on the expression of antioxidant enzymes, such as SOD, CAT, and APX, analyzed by native-PAGE and immunoblot assay (Figure 4 and Figure 5) illustrate the tight regulation of Si in ROS metabolism. Conversion of O_2_^−^ into H_2_O_2_ by SOD boon the rapid diffusion of H_2_O_2_ across the cell membrane [4,5]. Higher expression of CAT, a major constitutive enzyme on leaf glyoxysomes and peroxisomes directly scavenge the excessive H_2_O_2_. Additionally, the upregulation of APX detoxifies H_2_O_2_ into H_2_O with the utilization of ascorbate as a donor [4,5,6]. Integral coordination of antioxidant enzymes could be vital for the redox homeostasis mechanism under the oxidative stress in *R. hybrida* ‘Rock Fire’. Similar results were previously reported in wheat [34] and barley [35].

Addition of Si with NaCl restricted the Na accumulation in *R. hybrida* ‘Rock Fire’ (Figure 6A). In concordance with our findings, significant reduction of Na content was previously found in wheat [18], barley [26], cucumber [27], and tomato [32]. Deposition of Si on the inner tangential region of root endodermis reduces the transpirational bypass-flow of Na ions [25] and/or blocked with the formation of Na complexes [36]. Concurrently counter-uptake of K against Na was observed in the Si-supplemented NaCl treatment (Figure 6B). This competitive counteractivity could decrease the transpiration rate and/or activate the H-ATPase in the membrane [32,36]. Accumulation of other macro- and micro- nutrients was also modulated due to the Si and/or NaCl treatments. Either higher or lower content of other essential elements observed between the treatments instigate the involvement of Si in the metal homeostasis [37]. Macronutrients are essential constituents for the building blocks of plants. Similarly, micronutrients are indispensable components for the activation of enzymes and involved in signaling processes. Changes in the macronutrient status in the cell could facilitate various fundamental processes, such as photosynthesis, stomatal development, cell division, carbohydrate accumulation, pigments production, and the synthesis of nucleic acid and proteins. In the same way, modulations in the micro-nutrient content activate/repress several metabolic processes [38]. These fluctuations are indispensable according to the circumstances and Si appeared to be positively regulated the elemental uptake in *R. hybrida* ‘Rock Fire’.

Deterioration in the development (Figure 1) and impairment of the photosynthetic process (Figure 2) under salt stress conditions directly affected the expression of photosynthetic-related proteins (Table 1). A decline in the function of photosynthetic organelles decreased the efficiency of the plants to overcome stress [34]. Simultaneously, redemption of stomatal growth and stimulation of photosynthesis-related proteins denoted the involvement of Si in the major carbon fixation pathways, such as the Calvin cycle, tricarboxylic acid (TCA) cycle, and pentose phosphate pathway. Nwugo and Huerta also described the enhancement of photosynthesis related-proteins on rice plants treated with Si under cadmium stress [35]. NAD(P)H-quinone oxidoreductase subunit H (spot 7) and subunit J (24) are involved in the respiratory electron transfer chain and responsible for the oxidation of NADH [39,40]. NADH is a potential source of NAD^+^ and a principal electron donor to the respiratory chain reactions. Ratio of NADH/NAD^+^ is vital for the regulation of cellular pathways and ATP synthesis. Ferredoxin-NADP reductase (spot 12) is involved in the catalysis of photosynthetic electron transport from ferredoxin (Fe) reduced to NADP^+^ and is necessary for the assimilation of CO_2_ in plants. In addition, ferredoxin is integral for various metabolic processes, such as nitrogen fixation, phenolics biosynthesis, detoxification of xenobiotics, and biogenesis of iron-sulfur clusters [41]. Ferredoxin-NADP reductase is required for the electron flow and CO_2_ fixation [42]. Ribulose-1,5-bisphosphate carboxylase/oxygenase (RuBisCO) is the fundamental enzyme required for the fixation of CO_2_ during photosynthesis. Especially, the RuBisCO small subunit (spot 23) is necessary for carboxylation catalytic efficiency and CO_2_/O_2_ specificity [43]. In the biological membrane, photosystem I (PSI) is one of the largest multiprotein complexes and Ycf4 (spot 21, 28, and 30) are extrinsic thylakoid proteins; along with Ycf3, it is crucial for the assembly of PSI [44]. Higher accumulation of RuBisCO and Ycf4 proteins in the Si treatments ensures the photoprotection and improvement in the light-harvesting process for the physiological development of plants.

An increase in the expression of enzymes involved in carbon metabolism, such as β-glucosidases (spot 11) (involved in the hydrolysis of glycosidic bonds of β-d-glucosides and oligosaccharides to release glucose), β-galactosidases (spot 18) (catalyzes the cleavage of glycosidic bond of β-galactosides into monosaccharides), and glucose-1-phosphate adenylyltransferase large subunit (spot 30) (catalyzes the conversion of ATP + α-d-glucose 1-phosphate to diphosphate + ADP-glucose) implies the capability of Si to participate in starch and sucrose metabolism [45]. Similarly, the abundance of acetyl-CoA carboxylase (spot 13 and 21) a precursor enzyme for fatty acid synthesis involved in the carboxylation of acetyl-CoA into malonyl-CoA decreased in NaCl treatment alone was increased upon the Si inclusion. Acetyl-CoA carboxylase plays the major role in the fatty acid biosynthesis in plastids and various reactions in cytosol, such as the synthesis of flavonoids, anthocyanin, very long-chain fatty acids, malonylation of d-amino acids, and ethylene precursors [46]. Glycerol-3-phosphate dehydrogenase (GPDH) (NAD^+^) (spot 32), a major enzyme that bridges the carbohydrate metabolism and lipid biosynthesis has been decreased in the NaCl-treated plants. However the GPDH spot level was enhanced by Si addition. Activation of GPDH, especially under salt stress, could increase the yield of mitochondrial NADH [47], and also helps in the maintenance of redox potential in mitochondria [48]. Upregulation of proteins-related to energy metabolism mediated by silicate application in *R. hybrida* is in agreement with the previous report on *Lycopersicon esculentum* under salinity stress [32]. Hence, the induction of carbohydrate/fatty acid metabolism-related proteins ensures the proper maintenance of ATP and NADH or NADPH for cellular processes.

Modulations in the transcription/translation processes are the apparent phenomenon observed under any abnormal conditions. Protein spots, such as CTR9 homolog (spot 46), 50S ribosomal protein L14 (spot 25), and 60S ribosomal protein (spot 38), associated with transcription/translation were highly affected in the NaCl treatment. The CTR9 homolog plays a major role in the histone modifications during transcription [49]. The 50S ribosomal subunit functions as a catalyst in the peptidyl-transfer reaction of mRNA-directed protein biosynthesis [50]. The internal ribosome entry site recruited the mRNA with the interaction of 40S and 60S at the AUG codon to proceed with the elongation phase of translation [51]. Concordantly, a decrease in the abundance of the reactive intermediate deaminase A (spot 5), cysteine synthase (spot 39), and shikimate kinase 1 (spot 36) under saline conditions could affect the biosynthesis of amino acids, such as isoleucine, cysteine, phenylalanine, tyrosine, and tryptophan [52,53]. Inhibition of key-proteins required for the de novo synthesis of amino acids was in a reclamation state during Si treatments (Table 1). In addition, proteins associated with the terminal process of amino acid biosynthesis, such as ribosome-recycling factor (spot 16) and tRNA(Ile)-lysidine synthase (spot 22) downregulated under the NaCl conditions, were also improved upon the addition of Si. Reduction in the energy generation during the light-dependent reaction of photosynthesis reduces sulfate and nitrate, which are necessary for protein biosynthesis [35]. In particular, ribosome-recycling factor (spot 16) along with elongation factor-G catalyzes the breakdown of terminal complexes such as mRNA, tRNA, and ribosome [54]. Recycling of ribosome, a fundamental process for protein synthesis was enhanced by the Si. These enzymes play a key role in the protein maturation and its function. Ubiquitin conjugating enzyme E2 8 (spot 4) and E2 36 (spot 44), core proteins involved in the regulation of the ubiquitination pathway affected by NaCl that was recovered upon the addition of Si [55]. Possible Si-ubiquitin protein interactions under NaCl treatment could upgrade the protein regulation process during the post-translational modification and improves the protein specificity or selectivity. An increase in the abundance of transcription/translation-related protein presumably leads to the overall improvement on the cellular processes in the presence of silicate during both normal and stress conditions.

**Figure 10:**
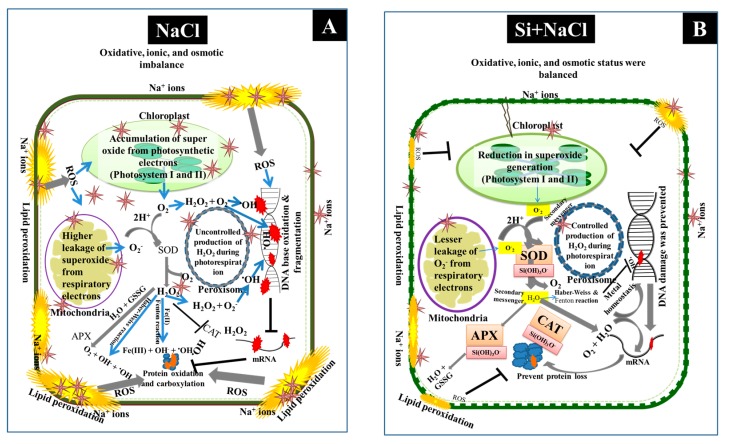
A possible simple and heuristic representation of oxidative damages occurred under salt stress conditions (**A**) and the amelioration of stress under Si supplementation by the induction of antioxidant enzymes and the prevention of nucleic acid and protein damages (**B**). The following are key for the symbolic representations provided in the figure: brown star, Na^+^ ions; red sparks, oxidation and fragmentation of DNA due to ·OH bound to DNA base; orange sparks, oxidation and carbonylation of proteins; grey arrow, normal or enhanced metabolism; black line with bar, reduction or inhibition; blue arrows, diffusion of ROS; green lines, cell wall layers including lipid bilayer; and small green squares on the membrane, polymerization of Si in cell wall.

Generally, production of ROS is markedly increased during oxidative stress. Excessive production of ROS during the salt stress condition predominantly depletes the crucial biochemical pathways and protein biosynthesis [32]. Glutathione *S*-transferase plays a major role in the breakdown of xenobiotics and it also plays a vital role in cell signaling [56]. Generally, plant peroxygenase is involved in the hydroxyl catalyzation of aromatics, sulfoxidations of xenobiotics, and oxidation of unsaturated fatty acids in an H_2_O_2_-dependent manner [57]. In a similar manner with our results (Table 1, spot 24), the induction of peroxidases were detected in rice seedlings exposed to Cd-stress [58]. As the minor levels of ROS are essential for cell signaling, a balance between the generation and detoxification of ROS were precisely maintained (Figure 10). The impact of silicate towards the reduction of oxidative damage caused by abiotic stress conditions is widely reported in several plants [18,19,20,25,26,27,28], including roses [30]. 

An increase in the abundance of anamorsin homolog (spot 7) under salt stress with Si supplementation could have improved the gas exchange, as the Fe-S protein groups play potential roles in the respiratory electron transport chain [59]. Metallothionein-like protein 4A (spot 39) involved in the homeostasis of essential metal and metal detoxification are necessary for the regulation of growth, proliferation, metalloenzymes activity, and stress response [60]. Ferritin-2 (spot 40) is an important protein in the iron homeostasis since it can store a large amount of iron in soluble, bio-available, and non-toxic form [61]. Therefore, the involvement of Si (Figure 7A)against stress is highly associated with its modulation on the essential elements uptake (Figure 7B–D) and ion-regulatory proteins (Table 1 and Figure 9A,B) regulate the nutrient availability and ion migration between the cellular compartments in the plants.

## 4. Experimental Section

### 4.1. Plant Materials, Treatments, and Growth Conditions

In vitro grown nodal explants of *R. hybrida* ‘Rock Fire’ were cultured in the Murashige and Skoog (MS) [62] medium with 1.0 mg·L^−1^ 6-benzyladenine (BA) and 0.5 mg·L^−1^ indole-3-acetic acid (IAA). All the cultures were maintained under a 16 h photoperiod provided by cool-white fluorescent light (60 μmol·m^−2^·s^−1^) at 25 ± 1 °C in the growth chamber. After three weeks, induced shoots were further sub-cultured in plant growth regulator-free MS medium containing 0.1% activated charcoal. Well-rooted young plantlets were acclimatized for three weeks in commercial medium (Tosilee Medium, Shinan Precision Co., Jinju, Korea). Totally, four treatments viz., control, 1.8 mM Si, 50 mM NaCl, and Si+NaCl were conducted hydroponically in a 375 mL magenta box (GA-7, Sigma Chemical Co., St. Louis, MO, USA) covered with foil. A bubble generator (BT-A65, PhilGreen, Seoul, Korea) was used for aeration with a time interval (1 min per hour). The nutrient solution was renewed every three days. Silicon was supplemented in the form of potassium silicate (K_2_SiO_3_). Excessive potassium was deducted from the potassium nitrate (KNO_3_) and the nitrate loss was balanced with the addition of nitric acid (HNO_3_) [20]. In total, ten containers for each treatment were arranged in a randomized block design. Immediately after harvest at 15 days, plants were frozen in liquid nitrogen and stored at –80 °C until further analysis. For all analyses three biological replicates were used.

### 4.2. Microscopy Observation and Pigment Analysis

After two hours of active photoperiod, the leaves were dissected from the plants. The leaf surface was gently peeled and fixed in slides with a few drops of glycerine [63]. Microscopic observation was performed at 40× magnification in a Nikon Eclipse Ci-L/S clinical microscope (Nikon Corporation, Minato-ku, Tokyo, Japan). Total chlorophyll and carotenoid contents were estimated by following the procedure of Sims and Gamon [64].

### 4.3. Reactive Oxygen Species and Lipid Peroxidation

For in situ histo-chemical localization of O_2_^−^ and H_2_O_2_, the third leaf from the plants were excised and placed in 4 mM riboflavin containing 25 µM nitro blue tetrazolium (NBT) (pH 6.4) for O_2_^−^ and 5 mM 3,3′-diaminobenzidine (DAB) in 10 mM phosphate buffer (pH 7.2) for H_2_O_2_ localization, respectively. Incubation was carried-out in a vacuum under dark conditions for 4 h. Finally, the leaves were boiled in ethanol (95%) until chlorophyll was washed away [65]. After fixing the samples in ethanol:glycerol:acetic acid (3:1:1, *v*/*v*), photographs were taken using a digital camera (PowerShot G10, Canon, Japan). Superoxide has been quantified based on Tian et al. [66]. H_2_O_2_ concentration was estimated by following the procedure of Gong et al. [67]. Lipid peroxidation level was determined by the concentration of MDA [27].

### 4.4. Analysis of Antioxidant Enzymes

#### 4.4.1. Activity and Native-PAGE Analysis of Antioxidant Enzymes

Samples for antioxidant enzymes activity analysis were prepared according to our previous report [20]. Superoxide dismutase (SOD) was assayed by nitro blue tetrazolium (NBT) inhibition method [67]. Catalase (CAT) activity was estimated based on the protocol of Cakmak and Marschner [68]. Activity of ascorbate peroxidase (APX) was determined by following the principle of Nakano and Asada [69]. Bradford assay was used to quantify the total soluble protein content [70]. Enzyme samples corresponding to 30 µg of protein were mixed with Laemmli buffer (6×) on 5:1 [71] and resolved on 6% stacking and 10% separating gel. Isozymes of SOD, CAT, and APX were identified according to the procedure of Shah and Nahakpam [72].

#### 4.4.2. Immunoblotting

For Western blot assay, 40 µg of protein from each treatment separated in 12.5% SDS-PAGE were electro-blotted on the polyvinylidene difluoride (PVDF) membrane. After blocking with 1× Tris-buffered saline (TBS) containing 5% skimmed milk, the membrane was incubated overnight at 4 °C in 1:500 dilutions of anti-SOD (Cell Signaling #2770 (Cell Signaling Technology Inc., Danvers, MA, USA), anti-APX (Cell Signaling #AS08 368), and anti-CAT (Cell Signaling #12980) antibodies, respectively. Incubation with secondary antibody conjugated with HRP anti-rabbit IgG (Cell Signaling #7074) were carried out in RT for 1 h. The blot was washed with 1 × TBS (3 × 5 min) before each step until the signal was detected. Finally, the signals were detected using chemiluminescence (Cell Signaling SignalFire ECL Reagent #6883) in a ChemiDoc MP System (BioRad, Hercules, CA, USA).

### 4.5. Analysis of Silicon, Sodium, and Potassium Uptake

The wet autoclave-induced digestion method was used to estimate the Si content [73]. Powdered, dried samples (100 mg) were mixed with 3 mL of 30% H_2_O_2_, 200 µL of octyl alcohol (for preventing excessive foaming), 4.2 g of NaOH, and made up the volume to 10 mL with distilled H_2_O. Vortexed samples were autoclaved at 110 °C for 1h. The autoclave-digested samples (100 µL) were mixed with 0.25 mL of 6N HCl, and 0.5 mL of ammonium molybdate solution (10%, pH 7.0). After incubation for 10 min, 0.5 mL tartaric acid (20%) was added and allow to stand for 5 min. Finally, 0.7 mL of reducing agent (1.2 M of sodium bisulfite freshly prepared containing 0.4 M of sodium sulfite and 0.335 M of 1-amino-2-napthol-4-sulfonic acid) were added and the volume was brought to 10 mL using distilled H_2_O. After 30 min, absorbance was read at 650 nm and the Si content was calculated from the reference standard curve using SiO_2_ (Sigma Chemical Co., St. Louis, MO, USA) as the standard. All the incubations were carried out at room temperature. Glass bottles and cylinders are avoided during Si estimation due to the presence of silica in the glassware. For measuring the nutrient content, samples were ashed at 525 °C for 4 h in the Naberthern muffle furnace (Model LV 5/11/B180, Lilienthal, Breman, Germany) and contents were measured using an inductively-coupled plasma (ICP) spectrometer (Optima 4300DV/5300DV, Perkin Elmer, Waltham, MA, USA).

### 4.6. Proteomics Analysis

#### 4.6.1. Protein Isolation

Leaf samples (100 mg) from each treatment were finely ground in liquid nitrogen and total protein was extracted by a modified phenol/ammonium acetate-methanol precipitation protocol [74]. Powdered samples were vortexed vigorously with the extraction buffer (100 mM Tris-HCl, 700 mM Sucrose, 2% sodium dodecyl sulfate (SDS), 5% β-Mercaptoethanol (ME), 10 mM ethylenediaminetetraacetic acid (EDTA), 10 mM dithiothreitol (DTT), and 2 mM phenylmethylsulfonyl fluoride (PMSF)). An equal volume of Tris-buffered phenol were added and vortexed again for 30 min at 4 °C. After centrifugation at 4 °C for 1 h at 18,000× *g*, the phenol extract was carefully recovered and mixed with a 1:5 volume of ice-cold 100 mM ammonium acetate prepared in 100% methanol and incubated for 20 min at –20 °C. The precipitated samples were centrifuged for 30 min at 12,000× *g* at 4 °C. The resulting pellets were washed with 100 mM ammonium acetate in 100% methanol containing 10 mM DTT and incubated at 20 °C for 20 min and centrifuged at 12,000× *g* for 20 min in 4 °C. Finally, extracts were vortex for 1 h at RT isoelectric focusing (IEF) buffer (8 M urea, 4% 3-[(3-Cholaminodopropyl) dimethyl ammonio-1-propanesulfonate (CHAPS) hydrate, 40 mM Tris, and 1.0% (*w*/*v*) bio-lyte (*pI* 3–10). The insoluble particles were removed by centrifugation at 12,000× *g* for 15 min at 4 °C.

#### 4.6.2. Isoelectric Focusing (IEF)

A total of 125 µL of re-swelling buffer containing 70 µg of protein was passively rehydrated for 15 h in a 7 cm immobilized pH gradient (IPG) strip (pH 4–7, GE Healthcare, Little Chalfont, Buckinghamshire, UK) in the IPGbox (GE Healthcare, Little Chalfont, Buckinghamshire, UK). The Ettan IPGphor 2 isoelectric focusing (IEF) unit (GE Healthcare) was used for one-dimensional electrophoresis. Focusing was done at 20 °C with 50 µA current limit per strip in four steps: 300 V for 0:30 (h:min) (step and hold), 1000 V for 0:30 (h:min) (gradient), 5000 V for 1:30 (h:min) (gradient), and a final step 5000 V for 0:36 (h:min) (step and hold). Total time taken until the final voltage of 8.0 KVh reached was 3:06 (h:min).

#### 4.6.3. Two-Dimensional Gel Electrophoresis and Silver Staining

After focusing, IPG strips were equilibrated (30 min) for reduction and alkylation in buffer (8 M urea, 2% SDS, 50 mM Tris-HCl (pH 8.8) 20% (*v*/*v*) glycerol) containing 1.0% DTT or 2.5% iodoacetamide, respectively. Proteins were separated in 12.5% SDS-PAGE (PROTEAN II Bio-Rad, Hercules, CA, USA) with 70 V for initial 10 min and 100 V until the dye-front reaches the end of the gel. Silver-stained images were documented using and EPSON (Seiko Epson Corporation, Shinjuku, Tokyo, Japan) high-resolution scanner.

#### 4.6.4. Protein Identification and Database Search

Protein spots from three individual replicates were analyzed by Progenesis SameSpots 2D software v4.1 (Nonlinear Dynamics, Newcastle, UK). Differentially-expressed proteins between the treatments on the spots showed more than 1.5-fold changes (*p* ≤ 0.05) in one way ANOVA analysis were considered for further analysis. Trypsin digestion and MALDI-TOF MS analyses were conducted according to Muneer and Jeong [28]. The protein function/name was obtained via MASCOT software [33] from SwissProt database. Gene ontology analysis was done in AgBase [75] for functional classification. A heat map plot was generated using GProX software to find the differential expression between treatments.

### 4.7. Statistical Analysis

All the data were subjected to one way ANOVA followed by Duncan’s multiple range tests at *p* ≤ 0.05, and an F-test was carried out to find the significance between treatments by using SAS software (Statistical Analysis System, V. 6.12, Cary, NC, USA).

## 5. Conclusions

Alleviation of salinity stress in *R. hybrida* with Si supplementation was evident from the present study. Amelioration of stress involved with the improvement of various factors, such as stomatal function, gas exchange, photosynthetic pigments, lipid peroxidation, controlled ROS generation, activity of antioxidant enzymes, and the abundance of proteins associated with vital regulatory functions. The ability of Si to induce the activities of antioxidant enzymes, such as SOD, CAT, and APX, could be the major phenomenon responsible for the detoxification of excessively-generated ROS. The restriction of Na and antagonistic uptake of K in Si+NaCl treatment correlated with the prevention of scorching and shriveling in leaves. Finally, the active involvement of Si on regulation of proteins involved in various metabolic pathways could facilitate a deeper understanding of the potential mechanism(s) adapted by plants to mitigate stress due to exogenous silicate supplementation. Therefore, the current endeavor concludes that the supplementation of Si enhances the physiological development and proteomic changes to render tolerance against salinity stress in *R. hybrida*.

## Figures and Tables

**Table 1:** Differentially-expressed protein spots identified from the two-dimensional gel electrophoresis of *Rosa hybrida* ‘Rock Fire’ leaf proteome.

Spot No. *^a^*	Accession Number *^b^*	Nominal Mass (M_r_) *^c^*	Theo./Exp. *pI ^d^*	Protein Identification	Species	Sc (%) *^e^*	Score *^f^*
Photosynthesis							
7	Q332S1	45577	5.43/5.47	NAD(P)H-quinone oxidoreductase subunit H	*Lactuca sativa*	84	40
24	A6MML0	18820	6.19/5.05	NAD(P)H-quinone oxidoreductase subunit J	*Dioscorea elephantipes*	39	47
12	Q7F8E8	40407	8.72/4.45	Ferredoxin-NADP reductase	*Oryza sativa Japonica*	18	20
10	Q945L0	9476	5.33/5.10	Cytochrome c oxidase subunit 6b-2	*Arabidopsis thaliana*	60	46
21	A9L9A7	9.59/5.40	9.59/5.20	Photosystem I assembly protein Ycf4	*Lemna minor*	36	40
28	A9L9A7	21440	9.59/6.15	Photosystem I assembly protein Ycf4	*Lemna minor*	36	40
23	P19312	20087	7.60/6.50	Ribulose bisphosphate carboxylase small chain SSU5B	*Lemna gibba*	28	34
26	P21218	43587	9.23/4.50	Protochlorophyllide reductase B	*Arabidopsis thaliana*	25	30
30	A7M975	21497	9.59/4.10	Photosystem I assembly protein Ycf4	*Cuscuta reflexa*	28	36
Energy metabolism							
9	Q7XKV5	60290	7.21/5.90	β-glucosidase 11	*Oryza sativa Japonica*	19	35
15	Q9SCV4	94293	8.09/5.10	β -galactosidase 8	*Arabidopsis thaliana*	9	41
3	Q9SE26	64295	7.28/4.00	Isocitrate lyase	*Dendrobium crumenatum*	20	40
25	P12300	56038	6.61/6.70	Glucose-1-phosphate adenylyltransferase large subunit	*Triticum aestivum*	16	32
27	Q8H2J9	47104	9.76/6.80	Glycerol-3-phosphate dehydrogenase (NAD^+^)	*Oryza sativa Japonica*	22	35
11	P85438	3396	9.99/4.10	Acetyl-CoA carboxylase	*Catharanthus roseus*	100	36
16	P85438	3398	9.99/5.10	Acetyl-CoA carboxylase	*Catharanthus roseus*	96	34
22	Q9LHH7	31589	5.77/6.55	Bifunctional protein FolD 2	*Arabidopsis thaliana*	38	51
Transcription/translation							
20	A4QK54	13682	9.39/4.55	50S ribosomal protein L14	*Arabis hirsuta*	54	38
32	Q9FF52	17946	9.02/5.15	60S ribosomal protein L12-3	*Arabidopsis thaliana*	51	34
40	B5X0I6	124486	5.20/4.45	Protein CTR9 homolog	*Arabidopsis thaliana*	15	36
5	Q94JQ4	19930	8.53/5.25	Reactive Intermediate Deaminase A	*Arabidopsis thaliana*	66	38
39	Q43317	34492	6.25/5.20	Cysteine synthase	*Citrullus lanatus*	40	40
36	Q5NTH4	33310	9.30/4.30	Shikimate kinase 1	*Oryza sativa Japonica*	34	45
14	A2YMU2	29709	9.35/5.10	Ribosome-recycling factor,	*Oryza sativa Indica Group*	23	33
17	Q32RJ9	49484	9.55/5.87	tRNA(Ile)-lysidine synthase	*Zygnema circumcarinatum*	13	32
Stress/redox homeostasis							
8	P23283	21940	8.91/4.00	Desiccation-related protein PCC3-06	*Craterostigma plantagineum*	38	33
18	Q08298	42658	9.42/4.60	Dehydration-responsive protein RD22	*Elaeis guineensis var. tenera*	20	28
37	P01053	9381	6.58/6.15	Subtilisin-chymotrypsin inhibitor-2A	*Hordeum vulgare*	38	27
19	Q40679	27454	6.00/4.00	Peroxygenase	*Oryza sativa Indica*	25	29
13	P30109	24081	5.54/6.80	Glutathione S-transferase PARB	*Nicotiana tabacum*	41	36
Ion binding							
2	Q9ZPX9	15339	4.34/6.80	Calcium-binding protein KIC	*Arabidopsis thaliana*	28	19
6	B6TB21	28001	5.20/4.45	Anamorsin homolog	*Zea mays*	48	30
31	O24475	72088	5.48/4.00	Pinene synthase	*Abies grandis*	14	28
33	Q0IMG5	8379	5.62/5.60	Metallothionein-like protein 4A	*Oryza sativa Japonica*	69	29
34	P29390	27863	5.75/4.31	Ferritin-2	*Zea mays*	23	36
Ubiquitination							
4	Q9FZ48	17277	6.74/4.47	Ubiquitin-conjugating enzyme E2 8	*Arabidopsis thaliana*	59	32
38	P35131	17277	6.74/4.60	Ubiquitin-conjugating enzyme E2 36	*Arabidopsis thaliana*	59	32
35	Q8GY23	404995	4.96/4.45	E3 ubiquitin-protein ligase	*Arabidopsis thaliana*	17	34
Putative							
1	Q9FKQ2	13622	8.53/5.38	Putative clathrin assembly protein	*Pinus koraiensis*	57	35
29	Q2R075	43258	6.62/5.55	Putative glutaredoxin-C11	*Oryza sativa Indica*	24	34

***^a^*** The spot no. corresponds to the numbers given in protein gel images (Figure 8); ***^b^*** Protein accession number determined by SwissProt database via the MASCOT software [33]; ***^c^*** Theoretical molecular mass (*M*r) calculated from MASCOT Peptide Mass Fingerprint; ***^d^*** Isoelectric point (p*I*) of spots identified from MASCOT Peptide Mass Fingerprint and protein gel images (Figure 8); ***^e^*** MASCOT score of protein hit; ***^f^*** Sequence coverage percentage.
